# A robust method for the amplification of RNA in the sense orientation

**DOI:** 10.1186/1471-2164-6-27

**Published:** 2005-03-01

**Authors:** Nicholas F Marko, Bryan Frank, John Quackenbush, Norman H Lee

**Affiliations:** 1 The George Washington University School of Medicine; 2 The George Washington University Medical Center, Department of Biochemistry and Molecular Biology; 3 The George Washington University Medical Center, Department of Pharmacology; 4 The Institute for Genomic Research (TIGR), Dana-Farber Cancer Institute, Department of Biostatistics and Computational Biology and Harvard School of Public Health, Department of Biostatistics

## Abstract

**Background:**

Small quantities of RNA (1–4 μg total RNA) available from biological samples frequently require a single round of amplification prior to analysis, but current amplification strategies have limitations that may restrict their usefulness in downstream genomic applications. The Eberwine amplification method has been extensively validated but is limited by its ability to produce only antisense RNA. Alternatives lack extensive validation and are often confounded by problems with bias or yield attributable to their greater biological and technical complexity.

**Results:**

To overcome these limitations, we have developed a straightforward and robust protocol for amplification of RNA in the sense orientation. This protocol is based upon Eberwine's method but incorporates elements of more recent amplification techniques while avoiding their complexities. Our technique yields greater than 100-fold amplification, generates long transcript, and produces mRNA that is well suited for use with microarray applications. Microarrays performed with RNA amplified using this protocol demonstrate minimal amplification bias and high reproducibility.

**Conclusion:**

The protocol we describe here is readily adaptable for the production of sense or antisense, labeled or unlabeled RNA from intact or partially-degraded prokaryotic or eukaryotic total RNA. The method outperforms several commercial RNA amplification kits and can be used in conjunction with a variety of microarray platforms, such as cDNA arrays, oligonucleotide arrays, and Affymetrix GeneChip™ arrays.

## Background

The increased use of microarray expression profiling to study both the molecular biology of cancer and the cellular physiology of difficult-to-isolate cell types has led to a growing need for methods that allow the use of limiting quantities of RNA. Small surgical biopsies, fine needle aspirates, cyto-lavages, punch biopsies and blood samples often yield only 1–4 μg quantities of RNA as starting material for expression profiling. This limitation has prompted the development of amplification methods that produce the quantities of RNA required for microarray analysis. Changing requirements for the type and quantity of amplified RNA, driven by evolving microarray technologies, have led to the development of novel amplification strategies. While current methods are capable of delivering high-yield RNA amplification, this is often only achieved after complex priming strategies (for example, involving 4 or more primers) are coupled with multiple rounds of PCR and/or *in vitro *transcription, resulting in time consuming and costly protocols. Here, we present an overview of RNA amplification strategies, identify key limitations to existing techniques, and describe a simple, robust, and cost-effective strategy for single round amplification of RNA in the sense orientation.

### RNA amplification methods

Early attempts to amplify RNA employed a strategy based upon the Polymerase Chain Reaction (PCR) [1-4]. These methods relied on the terminal transferase activity of reverse transcriptase to allow addition of primer sites to the 3' end of reverse-transcribed, first-strand cDNA. Multiple rounds of PCR primed from this site and from the poly-(A)^+ ^sequence on the second-strand cDNA could then be used to facilitate amplification. These methods were confounded by differential amplification of cDNA and by introduction of errors by *Taq *polymerase. This problem prompted the development of a linear, T7-based *in vitro *transcription (IVT) method by Van Gelder and Eberwine [5-7].

In what has now become known as the "Eberwine Method," RNA templates are primed with an oligo(dT) primer that has been 5' modified to contain a promoter for the T7 RNA polymerase and are subsequently reverse transcribed into first-strand cDNA. The RNA-cDNA hybrid is then treated with *E. coli *RNAse H, and priming for second-strand cDNA synthesis occurs by either RNA nicking and priming or cDNA hairpinning^6^. Second-strand cDNA synthesis is carried out with *E. coli *DNA polymerase and *E. coli *DNA ligase followed by blunt-ending with T4 DNA polymerase. Transcription and amplification are then accomplished using the T7 RNA polymerase, which binds to the T7 promoter introduced during first-strand cDNA synthesis, producing antisense RNA (aRNA).

Technical revisions of the Eberwine method have included changes in first-strand primer concentration to minimize the appearance of non-sequence dependent RNA in the amplified product [8], supplementation of second-strand priming with random primers to improve its efficiency, and modifications that allow multiple rounds of IVT to augment yield [6,9,10]. Concerns regarding the fidelity of amplification with these methods stem from the 3' bias introduced by the use of the promoter-modified oligo(dT) primer during first-strand cDNA synthesis, and questions remain over the degree to which this amplified RNA reflects the true transcriptome of the unamplified sample. To correct for this potential bias, three alternatives have been developed to the Eberwine protocol.

One such alternative [11] is based upon the Eberwine approach, but second and subsequent rounds of amplification are primed with random nonamer primers modified by the addition of an upstream T3 promoter sequence (T3N9 primer). IVT from this T3 promoter prevents serially compounding the 3' bias introduced by the oligo(dT) primer across multiple rounds of amplification. The T3N9 primer has also been used to prime the initial round of reverse transcription, a modification that is useful for amplifying partially-degraded samples of RNA [12]. In this case, the method sacrifices the ability to selectively amplify mRNA for the versatility generated by the random priming and subsequent amplification of any RNA sequence present in the sample.

A second alternative to the Eberwine method is the "template-switching" (TS) strategy [13]. This technique centers on the observation that the Moloney murine leukemia virus reverse transcriptase (MMLV-RT) adds an oligo(dC) region to the 3' end of first-strand cDNA after reaching the terminal end of an RNA template [14]. When an oligo(G) primer is added to the second strand RT reaction, it will hybridize with this oligo(dC) sequence, and the MMLV-RT will switch strands (the "template-switch") and continue the reverse transcription reaction. This strategy can be used to append a T7 promoter to the 5' end of the oligo(G) primer[13,15], facilitating RNA amplification by IVT. Yield can be further improved by combining this technique with PCR amplification after cDNA synthesis [16] for bulk production of amplified RNA [17]. Several variations on this theme involving changes in the primers and in the details of the PCR amplification have been described, all of which rely on a combination of TS primers and PCR-based amplification to produce large amounts of amplified RNA [18,19].

A third recently introduced alternative is Ginsberg's Terminal Continuation (TC) technique [20]. In this approach, the initial reverse transcription reaction is primed with a mixture of an oligo(dT) primer and a modified TC primer. The former primes the reverse transcription of mRNA, while the latter is essentially a T7 promoter-containing GC-rich sequence that primes second strand cDNA synthesis. According to Ginsberg, the "likely mechanism (for this incorporation) is that the TC primer binds preferentially to GC-rich CpG islands flanking 5' regions of DNA that contain promoter sequences." [21] Initial reports of the results of TC amplification show promise for linear amplification of high-quality RNA, but extensive validations of this method have yet to be conducted.

### Validity of amplified RNA in microarray applications

The degree to which the pool of amplified RNA generated by these methods reflects the unamplified sample from which it is derived is an obvious concern for microarray applications and other downstream analyses, and the history of attempts to validate RNA amplification methods is summarized by Nygaard et al. [22]. Briefly, they note that a combination of Northern blotting, dot blot differential screening of cDNA probes synthesized from aRNA, internal RNA standards, hierarchical clustering, qRT-PCR, subgroup analysis, and ratio-intensity (RI) plot analysis has lead to the conclusions that relative expression levels are well-preserved after 1–3 rounds of amplification, that important over- or under-expressed genes are detectable after amplification, and that amplification may actually improve detection of RNA present in low copy number. However, they noted that few studies quantitatively compare total RNA and amplified RNA, that the true sources of amplification biases have not been thoroughly investigated, and that the degree to which "noise" affects the differences in these profiles has not been adequately assessed. Accordingly, they conducted multiple hypothesis testing based upon t-tests and ANOVA analysis of several technical parameters to study the nature and the magnitude of biases and variability associated with the use of amplified RNA in microarray expression profiling. They observed that approximately 10% of genes showed statistically significant differences in relative expression level between amplified and their unamplified counterparts and noted that neither technical replication of amplifications nor molecular characteristics of the sample were the likely cause of these observed differences. Despite these differences, they stated that the increased quantity and purity of mRNA hybridized in studies using amplified RNA increases overall fluorescence intensity and improves detection of low-abundance messages. Because they noted that more that 50% of the amplified RNA showed log_2_(ratio) differences within ± 0.5 of the unamplified RNA, they concluded that RNA amplification is useful in expression profiling and is likely to assist in the measurement of low-abundance RNA.

Despite the methodological validation provided by these studies, only recently have statistically-based analyses been used to compare RNA amplification methods in a critical, head-to-head fashion. A study by Zhao [23] examined differences between T7-based amplification protocols, including the Eberwine method and the template switch (TS) technique, where these methods were used to amplify RNA extracted from tumor samples rather than from the idealized situation of cell lines. They observed that the use of TS strategies does not improve the fidelity of RNA amplification versus the Eberwine method, that there is good correlation between samples after amplification, that the overall bias introduced by T7-based amplification strategies is uniformly low, and that the results are reproducible. Their overall conclusion was that T7-based amplification protocols generate high-fidelity, amplified antisense RNA that is suitable for use in cDNA microarray analysis.

### Limitations of current amplification protocols

It is currently accepted that RNA amplification strategies based on the Eberwine method maintain relative mRNA levels between samples when amplifying from either total RNA or poly(A)^+ ^RNA [24,25], are useful with low microgram quantities of starting RNA [26], and are capable of preserving differential expression profiles when used in conjunction with microarray analysis [13]. Accordingly, efforts have become increasingly focused upon developing an optimized protocol that minimizes amplification bias, provides versatility, and reduces technical complexity. While current commercial kits and published protocols are making progress to these ends, we feel that several important dimensions have been overlooked, limiting their utility.

First, most commercial protocols produce RNA only in the antisense orientation. With the emergence of spotted oligonucleotide arrays and mixed cDNA/oligo arrays [27], appropriate orientation of amplified RNA is an important experimental consideration. While TS and TC protocols can be modified to produce sense RNA, this generally comes at the cost of increased technical and biological complexity but adds no demonstrable benefit over the Eberwine strategy. Second, many current protocols rely on multiple rounds of IVT and/or PCR to produce amplified RNA, and even a few rounds of this amplification has the potential to introduce sequence error [6] or systematic bias [28]. While some applications, such as laser capture microdissection, may produce only picogram quantities of RNA and thus require extensive amplification, it should be noted that even small biopsy samples frequently yield low microgram quantities of RNA and thus may require only a single round of amplification. Finally, the increasing complexity of many amplification protocols creates logistical problems when implementing them for large-scale projects. Protocols that require multiple rounds of amplification can accumulate material and labor costs that quickly exceed those associated with the actual microarray analysis, and amplification can become a "rate-limiting" (or "cost-limiting") factor in study design. Additionally, the number of enzymes, reagents, and custom primers continues to increase as protocols become more complex, a situation which is equally undesirable. We believe that a protocol that addresses these limitations and provides a versatile and robust method for RNA amplification is needed.

### Goals for a revised RNA amplification protocol

Our goal was to develop a strategy based upon the Eberwine method but with the ability to produce sense RNA from small quantities of total or poly-(A)^+ ^RNA extracted from both ideal samples (e.g. cell line RNA) and "real-world" samples (e.g. tumors or tissues). This protocol should avoid the need for PCR steps and should require a minimum number of primers (two). Additionally, the protocol should be cost-effective, efficient, and technically simple to conduct. Finally, the method should give results consistent with similar amplification techniques when used with subsequent microarray analysis. We believe these criteria have been met with our protocol, which consequently will be useful in a variety of laboratory applications.

## Results

### Priming efficiency and cDNA yield

Quantitation of first strand cDNA after hydrolysis of the cDNA/RNA hybrid shows that priming with the T7-Oligo(dT) promoter/primer followed by reverse transcription produces cDNA that totals 5–10% by mass of the original mass of total RNA (data not shown). This is consistent with the average percentage of mRNA in the total RNA sample and therefore suggests that priming proceeds efficiently and that first strand cDNA synthesis successfully copies the population of mRNA in the original total RNA sample. Quantitation of second strand cDNA after priming with the T3N9 promoter/primer indicates a 120% increase in mass versus the mass of the first strand cDNA (data not shown). This suggests that second strand priming proceeds efficiently and that the first strand cDNA is successfully converted to double stranded cDNA by the second strand synthesis reaction. The extra mass (in excess of 100%) is attributable to the use of random nonamer primers which initiates synthesis at multiple positions along each first-strand cDNA template.

### Median transcript length

While inherent constraints of reverse transcription limit the amount of truly "full-length transcript" produced during any RNA amplification scheme, it is nonetheless desirable for an amplification method to favor the production of long transcript. RNA amplified using our method has a median length of 794 nucleotides (range: 70–9000 nt) suggesting that this method is capable of preserving long transcript during amplification (Table 2).

**Table 2:** Yield and transcript length of amplified RNA produced by three amplification methods. Values in parentheses represent *p*-values from *t*-test comparing the value against that of the study protocol.

	**Study Protocol**	**RiboAmp Protocol (*p*-value vs. Study Protocol)**	**BDSMart Protocol (*p*-value vs. Study Protocol)**
Mean yield from 4 μg total RNA (, μg)	26.29	71.56 (<0.04)	4.87 (<<0.0001)
Median amplified RNA length (nt)	794	507 (<0.003)	764 (0.88)

### Quantitative yield

Our method produced an average () of 26.3 μg of amplified sense mRNA from 4 μg of total RNA (Table 2). Assuming that 5% of the original sample of total RNA was mRNA, this reflects a 130-fold amplification. The protocol has been tested with as little as 1 μg of starting total RNA and produces sufficient mRNA for microarray analysis (~2 μg) under these conditions (data not shown).

### Amplification bias

**Figure 2:**
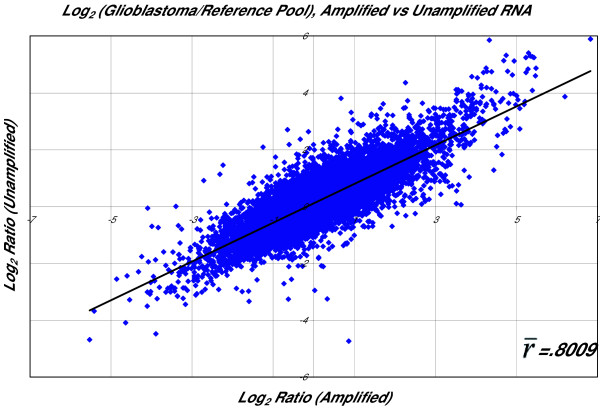
Correlation between expression profiles of RNA samples amplified using the study protocol and corresponding unamplified RNA samples demonstrates a low degree of amplification bias.

Any amplification will introduce some degree of bias into the population of amplified RNA. While recent reports suggest that the magnitude of this bias may be relatively unimportant as long as the bias is highly reproducible [29], we feel that aggressively limiting any source of experimental bias improves the quality of subsequent microarray data. We systematically evaluated the performance of RNA amplified using this protocol against identical hybridizations from unamplified RNA. Log_2_(ratio) plots of fluorescence intensity in amplified versus unamplified samples were constructed and analyzed for correlation as described above. Our results show that the amplification bias introduced by this method is small, as reflected in the high average correlation coefficient () between expression profiles from amplified and unamplified RNA samples ( = 0.8009, Table 3, Figure 2).

**Table 3:** Statistical analysis of microarray data generated by comparing hybridizations of amplified RNA samples to identical hybridizations of unamplified (total RNA) samples. Values in parentheses represent *P*-values from *t*-test comparing the value to that of the study protocol.

	**Study Protocol**	**RiboAmp Protocol (*p*-value vs. Study Protocol)**	**BDSmart Protocol (*p*-value vs. Study Protocol)**
Mean Correlation Coefficient ()	0.8009	0.7202 (<0.01)	0.7679 (0.23)
Mean % of elements with absolute difference of ± 2 log_2 _units	0.34	1.34 (<0.02)	0.60 (0.56)
Mean % of elements with absolute difference of ± 1.5 log_2 _units	1.31	4.05 (<0.05)	1.63 (0.67)
Mean % of elements with absolute difference of ± 2 log_2 _units	5.44	12.54 (<0.02)	6.52 (0.61)
Mean % of amplified array elements within ± 1 log_2 _unit	94.56	87.46 (<0.02)	93.48 (0.61)

### Reproducibility

**Figure 3:**
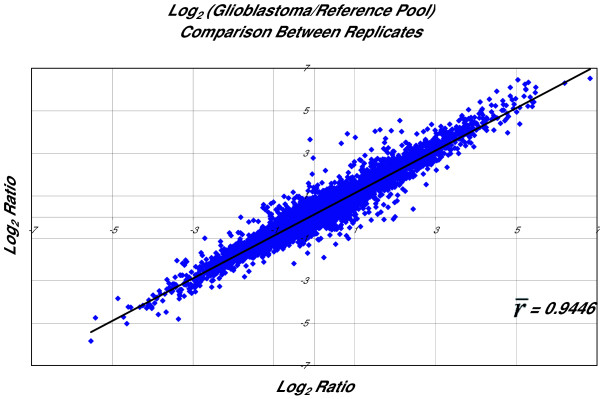
Correlation between independent replicates of expression profiles of RNA samples amplified using the study protocol demonstrates the high degree of reproducibility of the method.

We evaluated the reproducibility of this method and the consistency of the propagation of the amplification bias by comparing the expression profiles of hybridizations of three independently amplified RNA samples. Log_2_(ratio) plots of fluorescence intensity were constructed and analyzed as described above. Our results demonstrate a high degree of correlation between independent replicates ( =.9446, Figure 3) and a narrow dispersion (σ^2 ^= 0.00023, CV = 1.60%), suggesting that the amplification is highly reproducible and that the amplification bias is introduced consistently when the protocol is repeated.

### Comparison with commercial amplification kits

We compared these results to results from the RiboAmp mRNA amplification kit (Arcturus), which produces antisense RNA using a version of the Eberwine method, and from the BDSmart mRNA amplification kit (BD Biosciences), which produces sense RNA using a template-switching strategy. Both protocols were carried out according to the manufacturer's specifications using 4 μg of the pooled cell line RNA and the glioblastoma RNA as the starting material for amplification. The final yield of each method was measured using the NanoDrop spectrophotometer, the median size of the amplified mRNA from each kit was assayed using the Bioanalyzer 2100 (Agilent), and the amplification bias of each method was assessed using microarray analysis (as described above).

Three independent replicates were conducted for each method, and the reproducibility of the amplification was assessed as described above. The mean correlation coefficient () and the dispersion of each group (σ^2^, CV) were calculated, demonstrating a high degree of reproducibility for both the RiboAmp ( = 0.950, σ^2 ^= 0.0004, CV = 2.09%) and the BDSmart ( = 0.895, σ^2 ^= 0.0010, CV = 3.47%) protocols. These results suggest that subsequent comparisons of our amplification method to these commercial techniques are not confounded by individual variability among replicates. Additionally, the strong correlation between independent replicates of our method (see above) and of these commercial techniques suggests that performing additional independent replicates would not add significant statistical power to subsequent comparisons.

The average yield from our method ( = 23.6 μg) was less than that of the Arcturus RiboAmp method ( = 71.6 μg, p < 0.04) but greater than that of the BDSmart method ( = 4.87 μg, p = 1.7 × 10^-14^). The decreased yield versus the Arcturus RiboAmp method is expected, because IVT with the T7 promoter (production of antisense RNA, Arcturus method) proceeds more efficiently than IVT with the T3 promoter (production of sense RNA, our method). The improved yield over the BDSmart method is notable considering that both of these methods involve production of amplified sense RNA (Table 2).

The median length of the amplified RNA from our method (794 nt) was greater than that of the Arcturus RiboAmp method (507 nt, p < 0.003) and comparable to that of the BDSmart amplification method (764 nt, p = 0.88) (Table 2). The correlation between expression profiles of amplified and unamplified RNA was 10.6% better following amplification with our method ( = 0.8009) versus amplification with the Arcturus RiboAmp method ( = 0.7202, p < 0.01) and was comparable to the correlation after amplification with the BDSmart method ( = 0.7679, p = 0.15) (Table 3).

## Discussion

The emergence of methods to study the transcriptome initially necessitated the production of relatively large quantities of RNA from experimental systems, and Eberwine and Van Gelder's T7-based system of *In vitro *Transcription^5–6 ^became the first standard protocol for RNA amplification designed to fulfill these requirements. The evolution of microarray technology and other RNA techniques have imposed requirements on the type, length, and/or orientation of the starting RNA. Additionally, the growing interest in medical and microbial genomics now requires that data be collected from samples that are becoming progressively smaller and more difficult to acquire. This changing role for RNA amplification has catalyzed the development of multiple institutional and commercial amplification protocols, all of which claim to be high-yield, low-bias techniques capable of amplifying RNA with the specific characteristics required for downstream applications.

Template switching, terminal continuation, and other novel techniques for RNA amplification that have been developed over the past five years have become increasingly technically complex, and we feel that this is a significant disadvantage. In particular, they stray from the Eberwine approach, which has been extensively validated, and often rely on the use of proprietary (i.e. unspecified) enzymes, primers, and reaction components, which limit the utility of the methods (as well as protocol modification and fine-tuning). While proponents of these methods argue that the limitations are offset by improved yield, production of long transcript, reproducibility, and reduced amplification bias, we have found in practice that no single protocol is able to perform consistently in all four areas.

The technique that we have described affords the advantages of all of the aforementioned protocols while eliminating the major limitations and controlling the technical complexity. Several aspects of this protocol make it a robust tool that we believe will be useful in conjunction with a variety of experimental systems and downstream applications. First, the method we present is based upon the Eberwine technique. The methods are both straightforward and validated and do not require the use of custom enzymes, multiple (more than 2) proprietary primers, or PCR steps to complete the amplification. Second, this new method produces amplified mRNA in the sense orientation and can be used for production of aRNA with minor modification. This corrects for the downstream limitations imposed by antisense amplification in both the original Eberwine method and the Arcturus RiboAmp kit and allows amplified mRNA produced by our method to be used in conjunction with both cDNA (sense and antisense orientation) and long oligonucleotide (sense orientation) arrays. Third, our method requires only one round of amplification to produce RNA in the sense orientation. We believe that this represents a significant advantage over previously described, T7-based amplification strategies (most notably the method of Xiang [11]), where the modifications necessary for producing sense RNA require a minimum of two rounds of amplification. Fourth, our method produces sufficient amplified sense RNA for multiple microarray analyses after a single round of amplification from as little as 1 μg of total RNA. This yield is significantly higher than the BDSmart amplification kit and we have demonstrated that our method produces amplified mRNA that performs at least as well as the BDSmart amplification kit and superior to the Arcturus RiboAmp protocol in microarray applications.

Another advantage of our protocol is that it is readily adaptable to a variety of experimental systems. The approach that we have described synthesizes amplified RNA from eukaryotic total RNA in the sense orientation. However, because this method adds unique promoters to both the first and second cDNA strand (T7 and T3, respectively), either orientation of RNA can be produced from the same population of cDNA simply by selecting the appropriate RNA polymerase for IVT. Moreover, changing the two primers allows the protocol to be adapted to produce sense or antisense RNA from either prokaryotic or eukaryotic total RNA without any additional adjustments. The protocol can also be modified for use with either T3 or T7 IVT systems by changing the promoter sequence of the promoter-modified primer, it can be used to salvage partially-degraded RNA (as described by Xiang [11]), and it can be used for indirect RNA labeling protocols by substituting modified ribonucleotide bases into the IVT mix. Table 4 summarizes the primer pairs used for these alternate strategies, and Table 1 gives the necessary primer sequences. We have successfully tested a variety of these strategies and have achieved similar degrees of bias, reproducibility, and yield (data not shown). We believe that the ease with which this protocol can be adapted to a variety of experimental systems is a major advantage.

**Table 1:** Primer sequences for study protocol and its variations.

**Primer Name**	**Sequence**	**Concentration (ng/μL)**
T3N9	5'-GCGCGAAATTAACCCTCACTAAAGGGAGANNNNNNNNN-3'	100
T7N9	5'-GGCCAGTGAATTGTAATACGACTCACTATAGGGAGGCGGNNNNNNNNN-3'	100
Oligo(dT)_24_	5'-TTTTTTTTTTTTTTTTTTTTTTTT-3'	100
T3-Oligo(dT)_24_	5'-GCGCGAAATTAACCCTCACTAAAGGGAGATTTTTTTTTTTTTTTTTTTTTTTT-3'	100
T7-Oligo(dT)_24_	5'-GGCCAGTGAATTGTAATACGACTCACTATAGGGAGGCGGTTTTTTTTTTTTTTTTTTTTTTTT-3'	100
Random Hexamer	5'-NNNNNN-3'	3000

**Table 4:** Primer strategies for alternate versions of the study protocol.

**Type of Total RNA**	**First Primer**	**Second Primer**	**IVT System^†^**	**Type of Product**	**Orientation of Product**
**Eukaryotic**	Oligo(dT)_24_, T7-Oligo(dT)_24_	T3N9	T3	mRNA	Sense
	T3-Oligo(dT)_24_	Random Hexamer	T3	mRNA	Antisense*
Prokaryotic (or Partially Degraded**)	Random Hexamer	T3N9	T3	Total RNA	Sense
	T3N9	Random Hexamer	T3	Total RNA	Antisense

^† ^T7 IVT systems may be used by changing the promoter sequence of the modified primer to a T7 binding sequence

* Modified Eberwine Amplification

** As described by Xiang^11^

In clinical and research settings using laser capture microdissection or other strategies where very small amounts of starting RNA (10–100 ng) are available, multiple rounds of amplification may still be necessary. A final advantage of our protocol, as discussed previously, is that a second round of amplification can be easily incorporated and can be used to produce additional RNA in either the sense or antisense orientation. Future testing of this protocol will focus on the quantity and fidelity of the RNA population after multiple rounds of amplification and will include appropriate comparisons to commercial, multi-round techniques. However, the purpose of the present technique is to minimize bias while maintaining yield after a single round of amplification. In this setting, our results show that our method outperforms two commercial, single-step protocols when using identical quantities of initial RNA. We believe the performance and versatility of this method make it particularly beneficial to investigators attempting to conduct microarray analysis from 1–4 μg of initial RNA.

Neither this nor any other current strategy for RNA amplification is optimally suited to every experimental setting, and each technique has its own advantages and limitations. The challenge of selecting an amplification strategy is choosing a method that provides sufficient amplification and appropriate RNA orientation for downstream requirements yet minimizes the labor, cost, and bias introduced by the process. The method that we have described has been designed and validated for use in experiments where microarray analysis is to be performed with approximately 1 μg of starting total RNA. In this setting, our method produces a population of amplified RNA having minimal and consistent amplification bias, orientation compatible with both spotted cDNA and oligonucleotide arrays, and quantity sufficient for several downstream assays from a single amplification. Additionally, the protocol we present here can be easily adapted for use in a variety of experimental systems. This includes production of either sense or antisense RNA in prokaryotic or eukaryotic systems (simply changing one or both primers). None of these variations require multiple rounds of amplification to produce RNA of a specific orientation, but the protocol can easily be modified to enable multiple rounds when necessary.

Finally, we would like to note that there are some limitations to the method we have outlined here. Readers may note that several amplification strategies have been reported to produce greater than 1,000-fold increases in RNA yield, one aspect of our method that may be viewed as a limitation is its ~130-fold amplification. However, most higher-yield amplification strategies use multiple rounds of amplification and are subject to additional amplification bias. While we recognize that researchers using submicrogram quantities of starting material may consider strategies incorporating multiple rounds of amplification, in our experience even small tissue biopsies yield more than 1 μg of total RNA. The ability to amplify this quantity of RNA efficiently and effectively was one of our major design considerations. In this setting, 130-fold amplification produces more than enough RNA for multiple downstream microarray analyses and confers the benefit of reduced bias inherent in a single-round amplification protocol.

## Conclusion

We have developed a robust method for amplification of mRNA in the sense orientation. This protocol is based upon the Eberwine method and incorporates the advantages of more recent amplification techniques while eliminating many of the limitations of these strategies. Our method allows the production of sense mRNA with one round of IVT, yields 130-fold amplification, preserves long transcript, and produces mRNA that is well suited for downstream microarray applications. Microarray assays performed with RNA amplified using our protocol demonstrate that the method results in low amplification bias and is highly reproducible. Additionally, our method is readily adaptable for the production of sense or antisense, labeled or unlabeled RNA from intact or partially-degraded samples of prokaryotic or eukaryotic total RNA. The method outperforms several commercial RNA amplification kits and can be used in conjunction with a variety of downstream microarray platforms (cDNA arrays, oligonucleotide arrays, Affymetrix GeneChip™ assays). We feel that these advantages make our method a robust tool with the potential for application in a variety of research settings.

## Methods

### Overview of the method

The protocol that we have designed is based on the Eberwine method but uses novel priming strategies to produce amplified RNA in the sense orientation. First-strand cDNA synthesis from total RNA is primed with an Oligo(dT)_24 _primer followed by heat denaturing and then cooling to facilitate primer annealing. Reverse transcription produces cDNA-mRNA hybrids which are then subjected to alkali hydrolysis to remove template mRNA. First-strand cDNA (fs-cDNA) is separated from residual enzymes, nucleotides, and mRNA fragments using a spin column technique.

**Figure 1:**
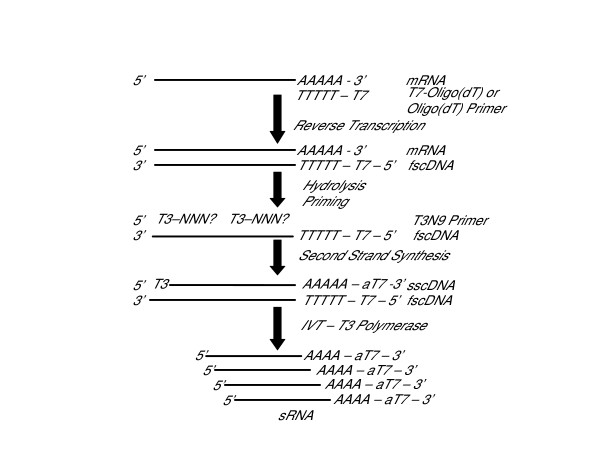
Overview of study method for amplification of RNA in the sense orientation

Second-strand cDNA synthesis is primed with random nonamers containing an upstream T3 promoter sequence (T3N9). The sample is heated to denature the fs-cDNA and to eliminate secondary structure. The temperature is rapidly dropped to the upper limit of the annealing range and then ramped more slowly to a final temperature of 4°C. We believe that this strategy minimizes "self-priming" of the fs-cDNA and, in the absence of competitive priming from RNA fragments, facilitates optimal annealing of the second-strand primer. Second-strand cDNA (ss-cDNA) synthesis is carried out using *E. coli *DNA polymerase and ligase followed by blunt-ending with T4 DNA Polymerase. The double-stranded cDNA is purified using a spin column technique, and *in vitro *transcription (IVT) from the T3 promoter sequences incorporated into the ss-cDNA produces amplified RNA in the sense orientation (Figure 1).

### Cell line RNA

Total RNA used in this study was derived from three cell lines: PA-1 ovarian teratocarcinoma, CaOV-3 ovarian adenocarcinoma, and U118MG glioblastoma. All cell lines were grown in a monolayer under Dulbecco's minimum essential medium (DMEM) with 10% fetal bovine serum (FBS) and 1% penicillin/streptomycin. Cell lines were held at 37°C in an atmosphere of 4% CO_2 _and maintained accordingly. When culture plates achieved ~90% confluence, RNA was extracted and purified following TIGR standard operating procedures based on the Trizol method (Invitrogen) [30]. RNA extracted from all three cell lines was pooled to create a RNA mixture with a final relative composition of 42.56% CaOV-3 RNA, 29.89% PA-1 RNA, and 27.55% U118MG RNA. Purity was verified by measurement of OD_260/280 _and OD_230/260 _in TE (pH 8.0) using a NanoDrop spectrophotometer, and RNA integrity was assessed by measurement of the 28S/18S ratio with the Agilent Bioanalyzer 2100 using the Agilent Total RNA Nano chip assay. The final values for the RNA pool were: OD_260/280 _= 2.04, OD_230/260 _= 2.20, 28S/18S = 2.13, indicating high purity, non-degraded RNA. Superas-In RNAse inhibitor (Ambion) was added to a final concentration of 1U/μL according to manufacturer's recommendations. RNA was stored at -80°C until it was used in the RNA amplification process.

### RNA amplification protocol

All temperature controlled steps in this protocol were conducted using a Peltier thermal cycler (PTC225 DNA Engine Tetrad, MJ research) in thin-walled, nuclease-free PCR tubes (BioRad). All enzymes, buffers, and other reaction components were purchased from Invitrogen unless otherwise noted. Four (4) μg of pooled total cellular RNA (described above) was as starting material for the amplifications. Priming for first strand cDNA synthesis is carried out by combining the total RNA with 1 μL of T7-Oligo(dT) primers (100 ng/μL, Ambion) and 1 μL of Superas-In RNAse inhibitor (20U/μL, Ambion). The mixture is diluted to 17.8 μL in DEPC treated water, heated at 70°C for 10 minutes, and cooled to 4°C for five minutes. First strand cDNA synthesis is accomplished by adding 6 μL of 5× First Strand Buffer, 3 μL 0.1M DTT, 1.2 μL of dNTP mix (50 mM), and 2 μL of SuperScript II reverse transcriptase followed by incubation at 42°C for 2.5 hours. After incubation, the reaction is cooled to 4°C for 2 minutes. Template RNA is hydrolyzed by adding 10 μL of 1N NaOH (Sigma) and 10 μL of 0.5M EDTA (Sigma) followed by incubation at 65°C for 15 minutes. The pH is subsequently neutralized by adding 10 μL of 1N HCl (Sigma). Hydrolyzed RNA and residual dNTPs are removed using the Minelute reaction cleanup kit (Qiagen) according to the manufacturer's protocol. Two rounds of elution from the column, each in 10 μL of Buffer EB (Qiagen) are performed to improve recovery.

Priming for second strand cDNA synthesis is accomplished by combining the eluted first-strand cDNA with 2 μL of random nonamer primers modified by the addition of a T3 promoter sequence at the 5' end (T3N9 primer, 100 ng/μL, Table 1)^11 ^(Operon) followed by incubation at 95°C for 3 minutes. Immediately following this incubation, the samples temperature is decreased as quickly as possible to 50°C followed by a temperature ramp from 50°C to 4°C at the rate of 0.4°C/s (~120s ramp time) and then a hold at 4°C for 2 minutes. Second strand cDNA synthesis is accomplished by adding 7μL of 5× Second Strand Buffer, 2 μL of dNTP mix (50 mM), 4 μL of *E. coli *DNA polymerase I (10U/μL), and 1 μL of *E. coli *DNA ligase (10U/μL) followed by incubation at 16°C for 2 hours. After incubation, blunt-ending is carried out by adding 2 μL of T4 DNA polymerase (5U/μL) and incubating at 16°C for 5 minutes. The reaction is terminated by adding 3.5 μL of 0.5 M EDTA (Sigma). Double stranded cDNA is purified using the Minelute reaction cleanup kit (Qiagen) following the manufacturer's protocol. Two rounds of elution with Buffer EB (Qiagen) are carried out to improve yield. The first elution is in 10 μL of Buffer EB, and the second is in 7 μL of Buffer EB. This provides the eluted cDNA in a final volume of 16 μL (corrected for 1 μL of retention on the column), which is the appropriate volume for use in the subsequent IVT reaction.

*In vitro *transcription is achieved using the T3 MegaScript kit (Ambion). A double reaction is used to improve yield, so 16 μL of NTP mix, 4 μL of 10× Reaction Buffer, and 4 μL of T3 MegaScript Enzyme Mix are added to the sample. The reaction is incubated at 37°C for 14 hours (overnight). The amplified RNA is purified using either the RNEasy Mini or the RNEasy Minelute kit (Qiagen) according to the manufacturer's protocol. Superas-In RNAse inhibitor (Ambion) is added to the amplified RNA to a final concentration of 1U/μL according to the manufacturer's recommendations.

### Assessment of products and intermediates

cDNA and RNA quantity and purity were assessed by measurement of OD_260/280 _and OD_230/260 _in the corresponding elution buffers (pH 8.0) using a NanoDrop spectrophotometer. RNA length distribution and integrity were assessed with the Agilent Bioanalyzer 2100 using the Agilent Total RNA Nano chip assay. Agilent's Bioanalyzer Expert 2100 software was used to quantify transcript length, and the software's automatic integration tool was used to determine the area under the curve. The median transcript length for each method was taken as the point at which half of the integrated area was achieved.

### Microarray analyses

Microarray analysis was conducted to determine the magnitude of amplification bias. Pooled human cell line RNA (as described above) as well as RNA extracted from one human glioblastoma were amplified as described. The amplified sense RNA (2 μg) was used as the starting material for synthesis of cDNA target, and a spiking control consisting of RNA transcripts of 10 genes from the Arabidopsis thaliana genome was added to each sample prior to cDNA synthesis in order to provide a consistent positive reference in subsequent hybridizations^28^. cDNA target synthesized from the cell line RNA was indirectly labeled with Cy-5 while target derived from the glioblastoma was labeled with Cy-3. The labeled cDNA from the cell line pool and from the glioblastoma were cohybridized to human 32,000 element spotted cDNA arrays. The array production, cDNA synthesis, target labeling, and hybridization were conducted following TIGR standard operating procedures [31]. Independent replicates were conducted for all amplifications and hybridizations.

In order to compare our method to commercially-available RNA amplification kits, microarray analysis was conducted using identical pooled cell line and glioblastoma RNA that was amplified with two commercial products (RiboAmp kit, Arcturus; and BDSmart mRNA Amplification kit, BD Biosciences). Amplification of 4 μg of total RNA was carried out according to the manufacturer's protocol for both kits, and 2μg of amplified RNA from each sample was used for microarray analysis as described above. Independent replicates were conducted for all amplifications and hybridizations.

Unamplified total RNA from both the pooled cell line and the glioblastoma was used as the starting material for the control arrays. Ten (10) μg of total RNA from each sample was used for cDNA synthesis, and the remainder of the protocol was carried out as described for the amplified samples.

### Data analysis

Microarray data was analyzed using the TIGR TM4 software package for microarray analysis [32]. TIGR Spotfinder was used to isolate spots on the arrays, correct for background, and assess the reliability of spots for downstream analysis. TIGR MIDAS was then used to adjust the data by applying LOWESS normalization [33,34] followed by standard deviation regularization [33,35]. The Cy5 channel (pooled cell line sample) was taken as the reference for all transformations. Normalized values of fluorescence intensity (FI) were log_2 _transformed, and  was calculated for each array element. Assessments of bias were conducted by comparing the log_2_(ratio) values for each element in the amplified sample arrays to their counterparts in the unamplified control arrays. Measurements of reproducibility were conducted by comparing the log_2_(ratio) values of corresponding elements in independent replicates.

### Statistical methods

The degree of bias introduced by an amplification method was assessed by plotting  against the  and calculating the correlation coefficient (*r*) for each independent replicate. The mean correlation coefficient () was calculated for each method, and differences between  for each amplification method were tested for statistical significance. Welch's T-Test [36] was used for all comparisons to control for the possible heteroscedastic nature of the original array data.

The degree of reproducibility of our amplification method was assessed by plotting the log_2_(ratio) values for paired hybridizations derived from independent amplifications of the same starting RNA, calculating the correlation coefficient (*r*) for a series of independent replicates, and computing the mean correlation coefficient (). Reproducibility was further investigated by calculating the variance (σ^2^) and the coefficient of variation  for  (CV), which describe the dispersion of the values contributing to .

## Authors' contributions

This method is based on an idea by Norman Lee. John Quackenbush and Nicholas Marko further developed the approach. Marko reduced this to practice in the laboratory, greatly expanding on the method and creating a robust protocol. Bryan Frank contributed significantly to the development, implementation, and optimization of the protocol. Marko conducted the data analysis and was largely responsible for the present manuscript.
